# Employability in the public sector: The impact of individual and organizational determinants

**DOI:** 10.3389/fpsyg.2022.1041618

**Published:** 2022-12-16

**Authors:** Brenda Vermeeren, Beatrice Van der Heijden

**Affiliations:** ^1^Department of Public Administration and Sociology, Erasmus University Rotterdam, Rotterdam, Netherlands; ^2^Institute for Management Research, Radboud University, Nijmegen, Netherlands; ^3^Faculty of Management, Open University of the Netherlands, Heerlen, Netherlands; ^4^Department of Marketing, Innovation and Organization, Ghent University, Ghent, Belgium; ^5^School of Business, Hubei University, Wuhan, China; ^6^Kingston Business School, Kingston University, London, United Kingdom

**Keywords:** personality, risk-taking behavior, transformational leadership, red tape, employability competences, internal employability, external employability

## Abstract

**Introduction:**

The importance of employability within organizations is increasing, due to various developments that initiate organizational changes. This study focuses on the employability in the public sector. While there seems to be a clear need for an employable public sector workforce, up until now there is little empirical research into the employability of workers in this sector, and into which specific individual and organizational characteristics influence it.

**Methods:**

We conducted structural equation modeling, using data from Dutch public sector employees (*n* = 13.471).

**Results:**

Our outcomes show that public sector employees consider themselves to be reasonably employable internally, and that they rate their external employability slightly higher. Moreover, it was found that both individual (personality and risk-taking behavior) and organizational characteristics (transformational leadership and red tape) influence their employability.

**Discussion:**

These results underline the dual responsibility of the employee and the organization in influencing workers’ employability within the public sector.

## 1 Introduction

The importance of employability within organizations is increasing, due to various developments that initiate organizational changes (e.g., globalization, technological progress and innovation, and demographic trends) (Fugate et al., 2021). Employability is the ability to perform the current job, to acquire a new job or to create work by making optimal use of existing competences (Van der Heijde and Van der Heijden, 2006). A high level of employability is seen as the responsibility of both organizations and their employees (Philippaers et al., 2019). In order to ensure that employees can continue to make a valuable contribution to the labor process, up until their retirement age, in a healthy manner and with a sense of well-being, research into employability is of great importance. This scholarly work has three contributions: (1) we examine employability specifically in the public sector context, (2) we focus on both perceived employability competences and internal and external employability, and (3) we study determinants of employability on the individual and on the organizational level in one and the same study.

As regards the first contribution, so far, only a few studies have examined employability in public organizations (Van Harten and Rodrigues, 2021; Van Harten and Vermeeren, 2021). Nevertheless, there are specific reasons that make workers’ employability an important issue for the public sector. Besides the general developments that initiate organizational changes, New Public Management (NPM) has come to play a central role within the public sector in recent decades, with values such as efficiency and effectiveness being emphasized (Osborne and Gaebler, 1992; Pollitt and Bouckaert, 2004; Boyne et al., 2006). Due to this business-oriented approach, strengthened by the economic crisis in the past years, many government organizations have been forced to adopt austerity measures and thereby to make changes in their organizational structures (Bozeman, 2010; Pandey, 2010; Raudla et al., 2013). At the same time, civil servants face new public service demands coming from an increasingly demanding society that is putting more emphasis on creating public value. Taken together, these changes call out for employable public sector workers, meaning that they need to adopt new roles and acquire new skills (OECD, 2017). The relevance of investing in workers’ employability in the public sector could furthermore be justified using the concept of public value, and by seeing investments in employability as a retention strategy (De Cuyper and De Witte, 2011; Rodrigues et al., 2020). More specifically, as these investments are likely to result in an increase in organizational commitment and intention to stay with one’s employer (ibid.), the public money that has been spent to these investments is valorized for the sector itself (i.e., return on investments). Retaining employable workers enables organizations to meet fluctuating demands for new products and services (Nauta et al., 2009). In this respect, employability provides a means for employers to match labor supply with demands in a changing environment (Thijssen et al., 2008).

Moreover, it should be noted that the public sector labor market has traditionally been different from that in the private sphere, and that its dominant practice in many countries is still lifetime employment (Bach and Bordogna, 2016) instead of lifelong employability (Thijssen et al., 2008). As a consequence, many public organizations in Western countries have even more elderly workforces than seen in the private sector. Public sector employers therefore need to manage workers’ employability so that they can remain active and productive during all, including the later, stages of their careers. This is even reinforced by the raising of the retirement age in most Western countries. So, while there seems to be a clear need for an employable public sector workforce, up until now there is little empirical research into the employability of workers in this sector, and into which specific individual and organizational characteristics influence it. Our study, therefore, aims to contribute to existing research through our focus on public sector employees.

As far as the second contribution of this scholarly work is concerned, there are quite some differences in the way in which researchers conceptualize and measure the concept of workers’ employability. Forrier et al. (2015) grouped different employability approaches into three categories. First, one group of researchers understand employability as an individual’s range of abilities and attitudes (personal strengths) necessary to acquire a job. This is also referred to as movement capital (Forrier et al., 2009). Examples of employability variables in this category are employability competences (Van der Heijde and Van der Heijden, 2006), up-to-date expertise (Van Harten et al., 2016), and a willingness to develop and change (Van Dam, 2004). Second, employability is sometimes regarded as the individual’s appraisal of available employment opportunities, in other words, their self-perceived job chances (Rothwell and Arnold, 2007; De Cuyper and De Witte, 2011). A third and less common perspective on employability addresses the realization of personal strengths and job chances, which is most noticeable when transitioning between jobs (Raemdonck et al., 2012). It is often assumed that these different notions of employability are interrelated (Forrier et al., 2015), but there is little scholarly work to confirm this. In our study, we include two of the three perspectives and look at whether an increase in workers’ employability competences (Perspective 1) is related to an increase in individuals’ perceptions of their employment opportunities (Perspective 2).

Knowing that employability has benefits, we posit that it is highly valuable to gain knowledge of its determinants, being the third contribution of our study. In previous scholarly work, a broad range of factors have been found to impact employability, and a review by Guilbert et al. (2016) categorized these into three groups of factors: (1) individual characteristics, (2) organizational characteristics, and (3) governmental and educational factors. In our empirical study, we focus on determinants on the individual and organizational level. In doing so, we adopt a multiple-stakeholder perspective on employability [see Fugate et al. (2021)] wherein the interaction between the individual and their employer form the basis for safeguarding both the workers’ career potential (i.e., their employability; Van der Heijde and Van der Heijden, 2006) as a contemporary form of job security, and as a viable means for strategically managing talent and a sustainable source of competitive advantage (Fugate et al., 2021).

In terms of individual characteristics, we focus on personality and risk-taking behavior. From earlier scholarly work in this field, we know that personality determines individual behavior in the workplace (Penney et al., 2011) to a considerable extent, and it appears to be an important predictor of work and career success as well (e.g., Seibert and Kraimer, 2001; Wille et al., 2013). And although previous research indicated that personality differs between public and private sector employees (Sudha and Khan, 2013), empirical work into the relationship between personality and employability of public sector employees is scarce so far.

Regarding employees’ risk-taking behavior, individuals who show a high degree of risk-taking are attracted to alternatives and seek out risks (Weber and Bottom, 1989) which might positively influence their employability. At the same time, previous research indicated that risk-taking behavior differs between public and private sector employees (Buurman et al., 2012) and to our knowledge, up until now empirical work into the relationship between risk-taking behavior and employability of public sector employees is absent at all.

Next to the impact of individual characteristics in the light of employability, which has received relatively more attention in comparison to the impact of organizational characteristics, in this study we also incorporate the role of leadership style and red tape (regulatory pressure), which we posit to be two important determinants in the public sector. Regarding leadership style, one’s direct supervisor plays a crucial role when it comes to maintaining and further promoting the employability of workers within an organization (Van der Heijden, 2005; Van der Heijden et al., 2017). As regards the second organizational factor, that is red tape, to the best of our knowledge, no research has been done into the relationship between red tape and employability yet. This is unfortunate as especially in research focusing on public sector employees, it is important to pay attention to the possible consequences of the ubiquitous amount of red tape (Steijn and Knies, 2021).

This paper is structured as follows. In the next section (see section “2 Theoretical framework”), we discuss the literature on employability competences and perceived employability in relation to its individual and organizational determinants. Based on these insights, our research hypotheses are formulated. Thereafter, we elaborate on the research method in Section “3 Methodology.” Subsequently, we present the outcomes of our analysis in Section “4 Results,” followed by a discussion of the findings and conclusions in Section “5 Conclusion and Discussion.”

## 2 Theoretical framework

### 2.1 Self-perceived employability competences and self-perceived job chances

In the scholarly literature, different approaches to employability can be distinguished, namely (1) an individual’s range of abilities and attitudes (personal strengths) necessary to acquire a job (movement capital), (2) an individual’s appraisal of available employment opportunities (self-perceived job chances), and (3) the realization of personal strengths and job chances, which is most noticeable when transitioning between jobs (job transitions) (Forrier et al., 2015). The assumption is that these different notions of employability are interrelated (Forrier et al., 2015), but more empirical evidence is needed to safely conclude. Following the notion of individual agency in the sustainable career framework (Van der Heijden and De Vos, 2015), the micro-level of the individual has become the core of attention in scholarly work on employability [see Fugate et al. (2021) for an historical outline]. In this study, we therefore look at the interrelatedness of two approaches to employability, namely at an individual’s range of abilities and attitudes (personal strengths) that are necessary to acquire a job (movement capital) on the one hand, and employee’s self-perceived job chances on the other hand. Before we discuss this relationship, we will first more clearly outline both approaches.

First, regarding the personal strengths or movement capital approach to employability, the competence-based operationalization of employability alludes to a person’s perception about their own abilities, capacities and skills that promote career possibilities (Van der Heijde and Van der Heijden, 2006), and which help them to maintain or enhance their functional, learning and career resources and skills (De Vos et al., 2011). This resource-based view clearly emphasizes personal agency as a mobilizer in achieving personal and career goals. In particular, Van der Heijde and Van der Heijden (2006) conceive competence-based employability as a positive resource for achieving beneficial career outcomes as well as for present and future performance.

Second, adopting a labor market perspective, employability can be regarded as the individual’s appraisal of available employment opportunities, in other words, their self-perceived job chances (Rothwell and Arnold, 2007; De Cuyper and De Witte, 2011; Vanhercke et al., 2014). Advocates of the notion of self-perceived employability argue that it captures the interplay between individual and contextual factors since people take both their individual capabilities as well as enhancing and hindering factors in their surrounding context into account when assessing their employability (Forrier et al., 2009; De Cuyper et al., 2012). An individual’s appraisal of employment opportunities can be perceived with their current employer (i.e., self-perceived internal employability) or with another employer (i.e., self-perceived external employability). Forrier et al. (2015) posited that the internal versus the external labor market are important foci in employability research that should be meaningfully integrated in one and the same study, herewith inspiring future researchers in this domain.

Combining the two perspectives and the two foci explained above in our scholarly work, and building on Forrier et al. (2015), we argue that competence-based employability can be regarded as an antecedent of self-perceived employability, because it enhances the individual’s perception of job opportunities. More specifically, competence-based employability plays the role of (1) foundation of the added value that each person perceives they have in the labor market; (2) motivational forces that energize and direct the search for new alternatives; and (3) dynamizer of the actions and decisions to respond to changes [see also Bargsted et al. (2021)].

Conservation of Resources (COR) theory (Hobfoll, 1989, 2001) provides a particularly useful framework to study the relationships between these employability perspectives (Bargsted et al., 2021), as it states that people strive for obtaining and protecting their resources (Hobfoll et al., 2018), defined as anything perceived as useful to attain personal goals (Halbesleben et al., 2014). Therefore, the acquisition of personal employability competences, such as the ones conceptualized and operationalized by Van der Heijde and Van der Heijden (2006), that improve work opportunities and aspirations, are relevant personal resources that help to maintain one’s job and boost career improvements. These authors defined employability as “continuously fulfilling, acquiring or creating work through the optimal use of competences” (p. 453). Their domain-independent operationalization consists of five dimensions, namely, (a) occupational expertise (domain-specific knowledge and skills), combined with four generic competences: (b) personal flexibility, meaning that one has the capacity to adapt easily to all kinds of changes in the internal and external labor market that do not pertain to one’s immediate job domain; (c) anticipation and optimization, that is, preparing for and adapting to future changes in a personal and creative manner and striving for the best possible results; (d) corporate sense, or one’s participation and performance in different work groups, such as organizations, teams, occupational communities and other networks; and (e) balance, which means compromising between opposing employers’ interests as well as one’s own (employee) opposing work, career, and private interests (ibid.).

Given the increasing need for a more flexible workforce in the public sector (Colley, 2001), in this empirical work, we focus on *occupational expertise*, being a domain-related competence, and on two flexibility-related dimensions, namely, *personal flexibility* and *anticipation and optimization*, being more generic employability dimensions (Van der Heijde and Van der Heijden, 2006). Personal flexibility is reactive and adaptive in nature, while the dimension of anticipation and optimization is a proactive and creative form of flexibility. Although the public sector, in comparison with the private one, is still characterized by a relatively higher job security and probability of long-term careers with one and the same employer (Clarke, 2017), its nature and structure are changing, and available resources (among others budgets) are decreasing (Van den Elsen et al., 2022). Altogether, this implies that public sector workers’ employability competences, in particular their knowledge and skills’ level and their capacities to adjust to all kinds of challenges at the workplace and in the broader labor market, are more and more relevant.

Using COR theory (Hobfoll, 1989, 2001) as our underlying framework, we propose that people who are in the possession of more employability competences, being a key personal resource at nowadays’ labor market, will perceive higher chances for obtaining, maintaining and/or improving their work positions and careers. Therefore, we hypothesize the following:

***H1:*** *The self-perceived employability competences of public sector employees are positively related to their internal (H1a) and external (H1b) employability.*

### 2.2 Employability determinants

Thijssen et al. (2008) stated that personal and contextual determinants are important factors influencing workers’ employability. In this study, we focus on two individual-level, personal determinants (i.e., personality and risk-taking behavior) and two organizational-level, contextual determinants (i.e., leadership style and red tape).

#### 2.2.1 Individual-level determinants

Focusing on individual-level factors that may foster one’s employability can help to explain why some employees are more employable than others (Fugate et al., 2021). An example of how personal attributes may contribute to the attainment of competences can be found in the work by Dweck and Leggett (1988). In their social-cognitive approach to motivation and personality, they describe the role played by the mind-set of people in their process of orientation toward certain goals (learning orientation vs. performance orientation) leading to adaptive or maladaptive behavioral patterns. In this section, we first discuss personality and then risk-taking behavior, being the two hypothesized personal determinants of employability that are taken into account in our study.

Personality theory proposes that the dynamic organization of mental structures and coordinated mental processes determines individuals’ emotional and behavioral adjustments to their environments (Allport, 1937, 1961; James and Mazerolle, 2001). Further, this theory states that there are recurring regularities or trends in psychological features – attitudes, emotions, and ways of perceiving and thinking – that exist inside a person that explain the recurring tendencies in an individual’s behavior (Hogan, 1991). As such, a central assumption is that personality determines individual behavior in the workplace to a considerable extent (Penney et al., 2011). In empirical research, personality has been found to be an important predictor of work and career success (e.g., Seibert and Kraimer, 2001; Wille et al., 2013). Wille et al. (2013) conducted a 15-year longitudinal study on perceived employability (referring to perceived job chances) and found that the Big Five traits (i.e., neuroticism, extraversion, openness, agreeableness, and conscientiousness) had substantial effects, even after controlling for a number of demographic and career-related characteristics, on perceived job chances. In a similar vein, Semeijn et al. (2020) studied the relationship between these personality traits and career success, and found significant associations between four of the five (agreeableness appeared not to be significant) personality traits and subjective career success outcomes. Following this line of reasoning and these empirical results, we assume personality to be related to public sector employees’ employability. Therefore, our second hypothesis is:

***H2:*** *Personality is related to both the employability competences (H2a) as well as to the internal (H2b) and external (H2c) employability of public sector employees.*

In addition to personality, in this study we pay attention to employees’ risk-taking behavior as a second individual-level determinant. Individuals who show a high degree of risk-taking are attracted to alternatives and seek out risks (Weber and Bottom, 1989). Individual risk-taking has a substantial impact on people’s decision-making processes and, consequently, on their career behavior (Yi and Wang, 2015). For instance, Plomp et al. (2019), in their study on job crafting and employability, found that in an environment of psychological safety people are more likely to be engaged in job crafting, and as a result enhance their employability. As psychological safety refers to employee perceptions regarding the consequences of interpersonal risk-taking (Edmondson, 1999; Baer and Frese, 2003), we posit that, just like the line of reasoning that Plomp et al. (2019) used to frame their scholarly work, individuals that portray more risk-taking behavior are more likely to engage in voice behaviors, initiative taking, and proactive behaviors (ibid.), all being important elements of job crafting.

Previous research indicated that risk-taking behavior differs between public and private sector employees with public sector employees being more risk averse than private sector employees (Buurman et al., 2012). However, to the best of our knowledge, up until now, empirical work into the relationship between risk-taking behavior and employability of public sector employees is absent at all. Building upon Plomp et al. (2019), we argue that in case people engage more in activities that are aimed at modifying their job tasks and/or relationships to create a better fit with their personal needs, goals and preferences (i.e., job crafting) (Wrzesniewski and Dutton, 2001; Tims and Bakker, 2010), they will develop more positive perceptions of their competencies and their ability to move between organizations (Chakraborty et al., 2017).

This leads to the third hypothesis:

***H3:*** *Risk-taking behavior is positively related to both the employability competences (H3a) of public sector employees as well as to the internal (H3b) and external (H3c) employability of public sector workers.*

#### 2.2.2 Organizational-level determinants

The second group of determinants in our study refers to organizational characteristics influencing employability. It is often argued that organizations have the responsibility to offer employees the support and facilities necessary to enhance their employability (Forrier and Sels, 2003; Thijssen et al., 2008). Although it is recognized that employers are often hesitant to support their employees in enhancing their employability, because of their fear of losing them to a competing organization (i.e., the employability or management paradox), various studies indicate that in case of resource investment by the employer a social exchange mechanism comes into play resulting in employees having a stronger intention to remain with their current employer (e.g., Nauta et al., 2009; De Cuyper and De Witte, 2011). Moreover, when employees perceive ample opportunities for internal employability or developmental opportunities to enlarge their existing competences within their organization, they also score higher on their level of self-perceived employability (De Vos et al., 2011).

Especially the direct supervisor plays a crucial role when it comes to maintaining and further promoting the employability of employees in an organization (Van der Heijden, 2005). Traditionally, HRM implementation was primarily the responsibility of HR professionals although, to some extent, line managers have always had some responsibility for HRM because they are held accountable for the work of their subordinates (McConville and Holden, 1999). However, the balance between line managers and HR specialists with respect to HRM implementation seems to have changed. There is clear evidence that, besides their traditional supervisory duties, line managers are increasingly expected to execute HRM activities as well, with many traditional personnel practices having been devolved to line managers (Purcell and Hutchinson, 2007). This seems particularly the case for employee development practices (Renwick and MacNeil, 2002). It is widely asserted that for career enhancement to be effective in organizations, line managers need to support the development of their staff and to have the necessary skills to coach and counsel them appropriately (Yarnall, 1998). In this respect, line managers influence their subordinates’ employability via supportive and inspirational behaviors, particularly those associated with transformational leadership (Van der Heijde and Van der Heijden, 2014; Xie et al., 2019).

Previous research showed differences between public sector and private sector employees in terms of their perceptions of transformational leadership, with public sector employees perceiving less individualized support (Top et al., 2015). However, scholarly work into the relationship between transformational leadership and employability is still scarce (Van den Elsen et al., 2022). Moreover, a growing body of research suggests that public managers operate within contexts that require rather distinctive skills and knowledge (Tummers and Knies, 2016) which emphasizes the importance of studying the influence of leadership within the public sector context. Previous private sector research has shown that transformational leaders influence employees’ attitudes and behaviors through individualized support and intellectual stimulation, which have been found to enhance their employability (Van der Heijden and Bakker, 2011; Böttcher et al., 2018). Building on this theoretical outline, the following hypothesis was formulated:

***H4:*** *A transformational leadership style is positively related to both the employability competences (H4a) as well as to the internal (H4b) and external (H4c) employability of public sector employees.*

The second organizational factor that we consider in this study concerns red tape. Red tape has been viewed as a key concept in public administration for decades, and one that can significantly impact the HRM process (Blom et al., 2021). A central facet of red tape is the presence or perception of rules that entail a compliance burden and that have no legitimate goal (Bozeman, 1993), which separates it from concepts such as formalization and green tape (Pandey and Scott, 2002; DeHart-Davis, 2009). To the best of our knowledge, so far no research has been conducted into the relationship between red tape and employability. Theoretically, red tape is argued to (a) constrain organizational practices, (b) alienate employees from their organization and, ultimately, (c) lower performance (Blom et al., 2021). Employees’ experiences of red tape, which is more prevalent in public than in private sector organizations, could hinder performance effects or further stimulate the turnover of employable individuals who perceive better job chances elsewhere (Quratulain and Khan, 2015; Shim et al., 2017). We hypothesize that the negative effects of red tape on employee satisfaction, and its positive influence on departure propensity and stress (Quratulain and Khan, 2015; Giauque et al., 2019), may also negatively affect workers’ employability. This leads to the fifth hypothesis:

***H5:*** *Red tape is negatively related to both the employability competences (H5a) and to the internal (H5b) and external (H5c) employability of public sector employees.*

**FIGURE 1:**
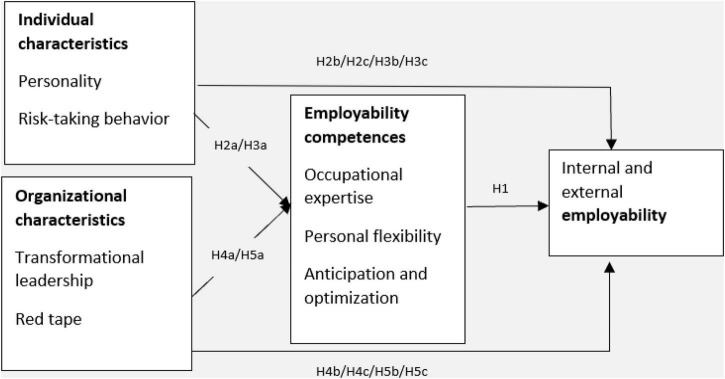
Research model.

Our research model (Figure 1) shows the incorporated individual and organizational characteristics as determinants of employability competences, which in turn are considered to influence internal and external employability.

## 3 Methodology

### 3.1 Sample and procedure

Data were collected within the Dutch public sector by the Central Bureau of Statistics on the behest of the Dutch Ministry of Interior and Kingdom Relations. In total, almost 95,000 employees (including 7,500 private sector workers) received an invitation letter to participate in the so-called Work Survey. In this letter, the respondents were guaranteed complete confidentiality and anonymity, and it was explained that the data were managed in accordance with the Dutch Personal Data Protection Act. In addition, this research was approved by the faculty’s ethics committee. The sampled respondents could complete the questionnaire online, and they received two reminders as encouragement. Responses were submitted between March 12th and June 2nd 2019. A total of 39,640 respondents replied, comprising a response rate of 41.7%. For our study, we only included the respondents without a supervisory position (*N* = 33,768) and from public administration sectors [central government (39%), regional government (13%), local government (31%), water boards (13%), and judicial authority (4%)] (*N* = 13,471). Employees from educational and health care subsectors were excluded.

In total 56.8% of the respondents were male. The respondents were asked to indicate their age group (0–24; 25–29; 30–34; 35–39; 40–44; 45–49; 50–54; 55–59; 60–64; ≥65), with the average corresponding to age group 6 (between 45 and 49 years old). Furthermore, respondents were asked about their highest completed education level (ranging from 1 = practical secondary school to 8 = bachelors/master’s degree from university or Ph.D.). The predominant educational levels were in group 7 and 8 which together covered 63.6% of the answers. The gender and age distributions in the participating sample are close to that of the total amount of public sector employees in the sectors included in our study, according to the Ministry’s employee information base.^1^ Although there are no official figures for the educational level distribution of these combined sectors, the available values for individual subsectors do not suggest that our sample is unrepresentative.

### 3.2 Measurement

First, we conducted an Exploratory Factor Analysis (EFA) on all latent constructs using the full data base with 39,640 respondents. The criteria used for item selection were: (1) only items with a factor loading of 0.4 or higher were included, (2) minimum of 3 items per construct unless the Cronbach’s alpha outcomes did not allow this, (3) one-dimensionality; and (4) Cronbach’s alpha of 0.7 or higher (Field, 2000). This exploratory factor analysis clearly indicated that the expected factor structure was found with our data. Based on the criteria, we only had to remove one of the items for the external employability measure.

We then conducted a Confirmatory Factor Analysis on the public administration data file selection (13,471 respondents). All factor loadings were significant and above 0.4. The model fit scores showed a good fit (NFI 0.93, CFI 0.94, and RMSEA 0.034). To further assess the convergent and discriminant validity of our constructs, we calculated the average variance extracted (AVE) for each of them. The AVE scores were above 0.50 for almost all variables (Fornell and Larcker, 1981), herewith showing satisfactory convergent validity. Exceptions were found for internal employability (0.45) and for occupational expertise (0.49). However, these scores appeared to be only slightly below the threshold, and all other scores did not give cause for any concern. To assess the discriminant validity of our concepts, we assessed the squared correlation coefficients between all constructs. For none of the constructs, this coefficient was higher than the AVE scores which demonstrates discriminant validity.

*Employability* was measured using the items developed by Rothwell and Arnold (2007). This scale split into internal (Cronbach’s alpha 0.76) and external employability (Cronbach’s alpha 0.83 – 1 item removed). An example item for internal employability is “I have good prospects in this organization because my employer values my personal contribution” and an example item for external employability is “I could get any job, anywhere, so long as my skills and experience were reasonably relevant.” The participants were asked to respond to the ten item statements on a five-point Likert scale (1 = fully disagree, 5 = fully agree).

The *employability competences* were measured using some of the items developed by Van der Heijde and Van der Heijden (2006). Respondents were asked about their perceived employability competences as previous research has shown that people act upon their own perceptions (Van Emmerik et al., 2012). Three dimensions of their employability competences were measured: occupational expertise (three items), personal flexibility (three items), and anticipation and optimization (four items). A sample item for occupational expertise is “In my work difficult tasks are given to me” (α = 0.72), for personal flexibility is “I am able to respond quickly to changes in my work environment” (α = 0.86), and for anticipation and optimization is “I plan further steps in my career” (α = 0.81). The participants were asked to respond to the ten item statements on a five-point Likert scale (1 = fully disagree, 5 = fully agree).

*Personality* was measured using the Big Five short-form 10-item measure by Rammstedt and John (2007) with 2 items for each of the five personality dimensions: neuroticism, extraversion openness, agreeableness, and conscientiousness. A sample item for neuroticism is “I am someone who gets nervous easily,” for extraversion is “I am someone who is outgoing, sociable,” for openness is “I am someone who has an active imagination,” for agreeableness is “I am someone who is generally trusting,” and for conscientiousness is “I am someone who does a thorough job.” The participants were asked to respond to the ten item statements on a five-point Likert scale (1 = fully disagree, 5 = fully agree).

*Risk-taking behavior* was measured using three single items. (1) “Would you dare to take a controversial position openly in a group?”, (2) “For your dream job, would you give up a permanent contract for a temporary one?”, and (3) “Have you ever quit your job before you got a new one?”. The participants were asked to respond 1 = no or 2 = yes to all three items.

*Transformational leadership* was measured using some items developed by Podsakoff et al. (1990). The respondents responded to six statements using a five-point Likert scale (1 = fully disagree, 5 = fully agree). A sample item is “My direct supervisor inspires us with his/her plans for the future.” Again, the Cronbach’s alpha was good (α = 0.86).

*Red tape* was measured using three items which has been used in previous research by Vermeeren and van Geest (2012) and Borst (2018) and were measured using a five-point Likert scale (1 = fully disagree, 5 = fully agree). The items were: (1) “It takes me a lot of time to comply with all the rules and obligations in my job,” (2) “Rules and regulations in my organization are more important than my experience,” and (3) “Rules and procedures in my organization make it difficult for me to do my job.” The Cronbach’s alpha was 0.81.

Finally, five individual-level control variables were included in our analyses: gender, age group, highest completed education, contract hours per week, and contract type. We dummy-coded gender (0 = male, 1 = female). Age was categorized in ten age groups: 0–24; 25–29; 30–34; 35–39; 40–44; 45–49; 50–54; 55–59; 60–64; ≥65. Education was measured on an eight-level scale (1 = practical secondary school to 8 = bachelors/master’s degree from university or Ph.D.). Contract hours was a continuous scale. Contract type was dummy-coded as 0 = permanent contract and 1 = temporary contract.

### 3.3 Analyses

First, the Exploratory Factor Analysis was conducted in IBM SPSS version 28. Second, the Confirmatory Factor Analysis was conducted using IBM AMOS version 28. Subsequently, we carried out structural equation modeling using IBM AMOS version 28 to test our research hypotheses.

This study was prone to suffer from common-source bias (CSB) because it fully relied on self-reported perceptual data (Podsakoff et al., 2012; Favero and Bullock, 2015). Despite such concerns, we used these data because employee perceptions and experiences are our key interest (George and Pandey, 2017). However, to properly address the possibility of CSB we conducted a Harman-1 factor analysis which showed no indication of inflated correlations in our data. The sums of squared% of variance was about 15%, which is well below the threshold of 50%.

## 4 Results

Table 1 shows the means (M), standard deviations (SD) and correlations between all model variables. The results show that public sector employees consider themselves to be reasonably internally employable (*M* = 3.26 on a five-point scale). However, they consider themselves more externally employable (*M* = 3.42 on a five-point scale) than internally employable. Regarding the employability competences, the results show that public sector employees feel that they have a reasonable amount of occupational expertise (*M* = 3.90 on a five-point scale) and personal flexibility (*M* = 3.90 on a five-point scale). The degree to which employees believe to be able to anticipate and optimize is on average a bit lower (*M* = 3.14 on a five-point scale) compared to the scores for occupational expertise and personal flexibility.

**TABLE 1:** Means (*M*), standard deviations (*SD*), and correlations.

	*M*	*SD*	1	2	3	4	5	6	7	8	9	10	11	12	13	14	15	16	17	18	19
1. Internal employability	3.26	0.69																			
2. External employability	3.42	0.79	0.433**																		
3. Occupational expertise	3.90	0.57	0.272**	0.367**																	
4. Personal flexibility	3.90	0.61	0.300**	0.303**	0.395**																
5. Anticipation and optimization	3.14	0.77	0.218**	0.408**	0.202**	0.307**															
6. Neuroticism	2.17	0.74	−0.094**	−0.154**	−0.303**	−0.334**	−0.075**														
7. Extraversion	3.36	0.84	0.142**	0.115**	0.119**	0.206**	0.116**	−0.135**													
8. Openness	3.51	0.80	0.073**	0.072**	0.111**	0.128**	0.133**	−0.022*	0.177**												
9. Agreeableness	4.03	0.61	0.110**	0.070**	0.086**	0.192**	0.038**	−0.124**	0.170**	0.171**											
10. Conscientiousness	4.04	0.70	–0.002	0.025*	0.114**	0.074**	0.000	−0.126**	0.038**	−0.080**	0.179**										
11. Risk-taking: take a controversial position	1.86	0.35	0.040**	0.093**	0.190**	0.145**	0.038**	−0.184**	0.200**	0.122**	−0.043**	−0.052**									
12. Risk-taking: give up permanent contract	1.49	0.50	0.089**	0.271**	0.081**	0.146**	0.307**	−0.035**	0.061**	0.075**	0.008	−0.093**	0.015								
13. Risk-taking: resign from job	1.21	0.41	–0.015	0.081**	0.052**	0.076**	0.103**	–0.019	0.044**	0.086**	0.000	–0.022	0.015	0.170**							
14. Transformational leadership	3.37	0.69	0.501**	0.119**	0.067**	0.146**	–0.003	−0.036**	0.055**	0.026**	0.114**	0.034**	0.018	−0.033**	0.004						
15. Red tape	3.03	0.88	−0.259**	−0.041**	0.037*	−0.136**	0.056**	0.056**	−0.031**	0.049**	−0.054**	−0.080**	0.038*	–0.011	0.005	−0.229**					
16. Gender (1 = female)	0.43	0.50	–0.008	−0.031*	−0.069**	0.021	0.108**	0.125**	0.123**	−0.023**	0.046**	0.138**	−0.152**	0.097**	0.094**	−0.018*	−0.154**				
17. Age	6.56	2.14	−0.201**	−0.345**	−0.052**	−0.120**	−0.402**	−0.099**	0.003	0.044**	0.012	0.079**	0.096**	−0.348**	–0.021	−0.036**	0.044**	−0.163**			
18. Educational level	6.41	1.72	0.104**	0.195**	0.210**	0.048**	0.193**	0.010	−0.031**	0.052**	−0.037**	−0.160**	0.032*	0.264**	0.026*	−0.026**	0.020*	0.064**	−0.217**		
19. Contract hours	33.30	6.47	0.065**	0.083**	0.134**	0.068**	0.059**	−0.091**	–0.017	0.009	−0.036**	−0.059**	0.091**	–0.002	−0.036**	0.009	0.070**	−0.321**	–0.014	0.042**	
20. Contract type (1 = flexible)	0.08	0.28	0.089**	0.161**	−0.064**	0.066**	0.168**	0.042**	0.006	–0.003	–0.008	−0.036**	−0.042**	0.193**	0.066**	0.028**	−0.047**	0.054**	−0.327**	0.074**	−0.023**

*p < 0.05; **p < 0.01.

Of the big five personality traits, conscientiousness has the highest average score (*M* = 4.04 on a five-point scale) in comparison with the other four personality traits (2.17 for neuroticism, 3.36 for extraversion, 3.51 for openness, and 4.03 for agreeableness). Regarding risk-taking behavior, employees score relatively high on daring to take a controversial position openly in a group (*M* = 1.86 on a two-point scale). About half of the respondents indicated that they are willing to give up a permanent contract in exchange for a temporary contract for a dream job (*M* = 1.49 on a two-point scale). A small proportion of respondents indicated that in the past there was a situation wherein they already had quit their job before finding a new one (*M* = 1.21 on a two-point scale). Finally, on average, employees experience transformational leadership (*M* = 3.37 on a five-point scale) and red tape (*M* = 3.03 on a five-point scale) to a reasonable extent.

**FIGURE 2:**
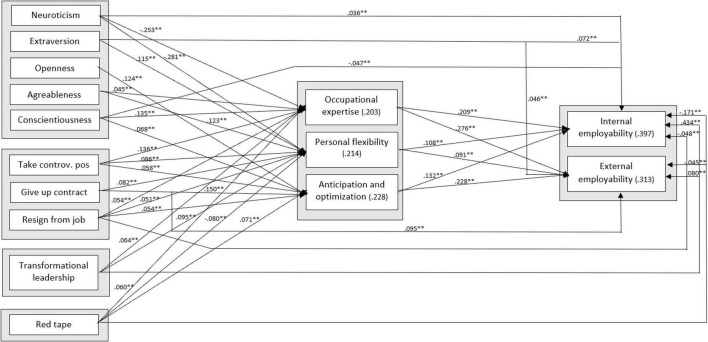
Structural equation model. **p < 0.01.

To test our research hypotheses, we conducted structural equation modeling. The outcomes of this structural model indicated an appropriate fit: NFI = 0.90, CFI = 0.91, and RMSEA = 0.037. In Figure 2, the overall results are presented. Only the statistically significant relationships are presented (with a significance level of 0.05). The numerical scores on all lines indicate standardized regression coefficients (β), and the scores in brackets are the explained variances. The control variables (gender, age group, highest completed education, contract hours per week, and contract type) are part of this tested model, but are for reasons of parsimoniousness not presented in the figure. These results can be found in Table 2.

**TABLE 2:** Standardized regression weights.

			β	*P*
Occupational expertise	<—	Gender	–0.028	0.045
Anticipation and optimization	<—	Gender	0.064	***
Internal employability	<—	Gender	–0.048	***
External employability	<—	Gender	–0.094	***
Occupational expertise	<—	Age	–0.083	***
Personal flexibility	<—	Age	–0.122	***
Anticipation and optimization	<—	Age	–0.333	***
Internal employability	<—	Age	–0.095	***
External employability	<—	Age	–0.186	***
Occupational expertise	<—	Educational level	0.219	***
Anticipation and optimization	<—	Educational level	0.073	***
External employability	<—	Educational level	0.032	0.008
Occupational expertise	<—	Contract hours	0.086	***
Personal flexibility	<—	Contract hours	0.048	***
Anticipation and optimization	<—	Contract hours	0.066	***
Occupational expertise	<—	Contract type	–0.085	***
Anticipation and optimization	<—	Contract type	0.029	0.013
Internal employability	<—	Contract type	0.025	***
External employability	<—	Contract type	0.056	0.034

***p < 0.001.

First, we discuss the results of the control variables in Table 2. Gender (1 = female) appears to have a negative relationship with occupational expertise and with both internal and external employability, and a positive relationship with anticipation and optimization. Furthermore, the outcomes of our analyses show a negative relationship between age on the one hand, and all three of the incorporated employability competences, and both internal and external employability on the other hand. Educational level appears to be positively related to occupational expertise, anticipation and optimization, and external employability. Contract hours are positively related to all three included employability competences. Finally, contract type (1 = temporary) is negatively related to occupational expertise, and positively related to anticipation and optimization, and to both internal and external employability.

Hypothesis 1 stated that the self-perceived employability competences of public sector employees are positively related to their internal (H1a) and external (H1b) employability. The results in Figure 2 show that all three employability competences are indeed positively related to both internal employability (occupational expertise β = 0.209, *p* < 0.001; personal flexibility β = 0.108, *p* < 0.001; anticipation and optimization β = 0.132, *p* < 0.001) and external employability (occupational expertise β = 0.276, *p* < 0.001; personal flexibility β = 0.091, *p* < 0.001; anticipation and optimization β = 0.228, *p* < 0.001) thereby fully supporting Hypothesis 1a and 1b.

Regarding the second hypothesis, that personality is related to both the employability competences (H2a) as well as to the internal (H2b) and external (H2c) employability of public sector employees, the results in Figure 2 show a statistically significant relationship between one or more of the personality dimensions and all three employability competences, thereby providing ample support for Hypothesis 2a. More specifically, neuroticism is negatively related to occupational expertise (β = −0.253, *p* < 0.001) and to personal flexibility (β = −0.281, *p* < 0.001). Extraversion is only positively related to personal flexibility (β = 0.115, *p* < 0.001). Openness is only positively related to anticipation and optimization (β = 0.124, *p* < 0.001). Agreeableness is positively related to occupational expertise (β = 0.045, *p* < 0.001) and to personal flexibility (β = 0.123, *p* < 0.001). Finally, conscientiousness is positively related to occupational expertise (β = 0.135, *p* < 0.001) and anticipation and optimization (β = 0.068, *p* < 0.001).

As regards Hypothesis 2b, the results show a significant relationship between three of the five dimensions of personality and internal employability, thereby confirming our expectation. In particular, neuroticism (β = 0.036, *p* < 0.01) and extraversion (β = 0.072, *p* < 0.001) are positively related with internal employability, while conscientiousness (β = −0.047, *p* < 0.001) is negatively related with internal employability.

Finally, Hypothesis 2c, which assumes a positive relationship between personality and external employability is partly confirmed as well. In particular, the results of our analyses indicate that extraversion has a positive relationship with external employability (β = 0.046, *p* < 0.001).

Our third hypothesis was that risk-taking behavior is positively related to both the employability competences (H3a) as well as to the internal (H3b) and external (H3c) employability of public sector workers. The results in Figure 2 show that the higher the willingness to take risks, the higher the scores on the employability competences, thereby supporting Hypothesis 3a. Employees who dare to take a controversial position openly in a group score higher on all three employability competences (occupational expertise β = 0.136, *p* < 0.001; personal flexibility β = 0.086, *p* < 0.001; anticipation and optimization β = 0.058, *p* < 0.001). Furthermore, employees who are willing to give up a permanent contract in exchange for a temporary contract for a dream job score higher on personal flexibility (β = 0.082, *p* < 0.001) and on anticipation and optimization (β = 0.150, *p* < 0.001). Finally, employees who indicated that they had already quit a job before finding a new one scored higher on all three employability competences (occupational expertise β = 0.054, *p* < 0.001; personal flexibility β = 0.051, *p* < 0.001; anticipation and optimization β = 0.054, *p* < 0.001). Altogether, with these outcomes Hypothesis 3a is strongly supported.

The results of our analyses do not show any support for Hypothesis 3b, which stated a positive relationship between risk-taking behavior and internal employability. On the contrary, our results indicated that employees who reported that they had already quit a job before finding a new one scored lower on internal employability (β = −0.048, *p* < 0.001).

Hypothesis 3c about the positive relationship between risk-taking behavior and external employability is partly supported with these data. Those who would be willing to give up a permanent contract for a temporary contract score higher on external employability (β = 0.095, *p* < 0.001).

Hypothesis 4 stated that a transformational leadership style is positively related to both the employability competences (H4a) as well as to the internal (H4b) and external (H4c) employability of public sector employees. The results presented in Figure 2 show that Hypothesis 4a is largely confirmed with these data. More specifically, there is a positive relationship between transformational leadership, on the one hand, and both occupational expertise (β = 0.064, *p* < 0.001) and personal flexibility (β = 0.095, *p* < 0.001), on the other hand. Remarkably, there is no significant relationship between transformational leadership and anticipation and optimization.

Hypotheses 4b and 4c are fully confirmed in this study. There is a positive relationship between transformational leadership, on the one hand, and both internal (β = 0.434, *p* < 0.001) and external employability (β = 0.080, *p* < 0.001), on the other hand. In particular, when a manager displays more transformational leadership, employees experience higher internal and external employability.

Finally, we expected red tape to be negatively related to both the employability competences (H5a) and to the internal (H5b) and external (H5c) employability of public sector employees. The results are presented in Figure 2. With regard to Hypothesis 5a, it is striking that there is a positive relationship of red tape with occupation expertise (β = 0.060, *p* < 0.001), and with anticipation and optimization (β = 0.071, *p* < 0.001), while there appears to be a negative relationship with personal flexibility (β = −0.080, *p* < 0.001). Therefore, there is just weak support for Hypothesis 5a with our data.

Furthermore, in line with Hypothesis 5b, red tape appears to have a negative relationship with employees’ internal employability (β = −0.171, *p* < 0.001). In other words, if people experience more red tape, they consider themselves less employable within their own organization. In a similar vein, red tape also appears to have a negative relationship with external employability (β = −0.045, *p* < 0.001), herewith confirming Hypothesis 5c as well.

## 5 Conclusion and discussion

In view of the increased aging of the population and the related labor shortages, which can be observed to a great extent in the public sector, and the speed at which changes (e.g., globalization, technological progress and innovation, and demographic trends) (Fugate et al., 2021) are taking place, paying attention to the employability of public sector workers is crucial. After all, public service delivery strongly depends on the employability of public sector employees.

Therefore, this scholarly work was aimed to examine both employability competencies and perceived internal and external employability, and their individual- and organizational-level determinants in the public sector context. Given the lack of empirical research on employability in public organizations, the first contribution of our work is that with our approach we partly close an important gap in the public sector literature. This is more and more important given the increasing and changing public service demands and skills’ needs, and the graying of the public sector population. The latter urges management to protect and ideally further enhance their employability, also in their later career stages.

As regards the second contribution of our work, building on Forrier et al. (2015) who called for more research integrating different notions of employability, both employability competences (i.e., employability operationalized as movement capital; Perspective 1) and perceptions of one’s internal and external employability opportunities (i.e., employability operationalized as self-perceived job chances; Perspective 2) have been incorporated in one and the same study. Last but not least, in line with the notion of sustainable careers (De Vos et al., 2020) which posits that both the individual career holder and their surrounding context, in particular their employer, are key stakeholders who need to safeguard workers’ employability, both individual-level and organizational-level determinants are taken into account. Adopting such a systemic or multiple-stakeholder perspective (Colakoglu et al., 2006) is an important third contribution of our study. After all, the challenges surrounding sound employability management encompass a dual responsibility, and it is required that over and above individual career management, other parties, such as one’s employer are actively involved [see also Van der Heijden (2005)].

The outcomes of our study show that public administration employees are reasonably internally and externally employable so there is certainly room for improvement. This result supports our approach to gain more insight into the factors that can influence employability. In particular, our results indicate that the three employability competences (occupational expertise, personal flexibility, and anticipation and optimization) are all related with the internal and external employability of employees in public administration. Herewith, this study is one of the first providing empirical evidence for the interrelatedness between different notions of employability, i.e., movement capital on the one hand and self-perceived job chances on the other hand. In other words, people who are in the possession of more employability competences, being a key personal resource at nowadays’ labor market, indeed perceive higher chances for obtaining, maintaining and/or improving their work positions and careers.

Furthermore, this empirical study shows that both individual and organizational characteristics influence the employability of public sector employees. This finding underlines the dual responsibility of the employee and the employer (Philippaers et al., 2019) (in this case, the immediate supervisor as well as the employer) with regard to monitoring and further promoting employability competences through this workers’ perceptions about employment opportunities (both within and outside the organization). The individual characteristics (personality and risk-taking behavior) are mainly related to the employability competences, whereas the organizational characteristics (transformational leadership and red tape) appear to play a major role in the internal and external employability of public sector workers.

A notable result is the negative relationship between risk-taking behavior and internal employability. Our results indicated that employees who reported that they had already quit a job before finding a new one scored lower on internal employability. This is counter-intuitive in our opinion, as one would expect that people are not inclined ‘to give up what they have,’ especially not in case they lack trust in their knowledge and skills that are needed in nowadays’ labor market. At the same time, our assumption was confirmed in case one’s external employability was the outcome measure. In addition, this study shows quite large differences in the degree of risk-taking behavior, depending on the aspect one looks at, and the results show that the relationships of the three risk-taking items with employability competences and internal and external employability are also different. So, it seems to matter which aspect of risk-taking behavior you look at in relation to employability of public sector employees. Altogether these results call for more research, but above all it endorses the importance of distinguishing between internal and external employability in one and the same study, as also posited by Forrier et al. (2015).

Of the organizational characteristics, a transformational leadership style has a positive relationship with two of the three employability competences and with internal and external employability which emphasizes the important role of the supervisor (Van der Heijden, 2005) when we consider workers’ employability in the public sector context. Remarkably, there is no significant relationship between transformational leadership and anticipation and optimization. This might indicate that managers are mainly occupied with stimulating the employability competences of employees in the current situation, and are less focused on influencing the competence of employees to prepare for and adapt to future changes, i.e., being proactive. This outcome is fully in line with previous research by Van der Heijden and associates and referred to by the concept of instrumental leadership (Van der Heijden, 2005), being leadership that is focused on the here-and-now and less future-oriented, instead of appropriate people management that also pays core attention to employees’ career sustainability over time (De Vos et al., 2020) [see also Boerlijst et al. (1993)].

In line with our assumptions, red tape is negatively related to both internal and external employability. One striking result is that red tape shows a positive relationship with two of the three employability competences, namely with occupational expertise and with anticipation and optimization of employees. More research is needed to clarify this outcome. It could be that the specific content of the regulatory burden plays an important role. Based on our research, it seems that some rules can indeed lead to an increase in employees’ competences, i.e., can have positive effects. Since, to the best of our knowledge, this is the very first study to examine the influence of red tape on employability, we recommend that follow-up research distinguishes between different types of rules, for instance, rules that are experienced as a major stressor/obstacle versus challenging rules or in other words by making a distinction between red tape (ineffective rules) and green tape (effective rules) and the way in which these rules are implemented within the organization (DeHart-Davis, 2009).

### 5.1 Limitations and future research

First of all, our research design is cross-sectional, which means that we are unable to establish evidence of a causal relationship between the variables under study. Longitudinal data is needed to better understand the causal and long-term effects of individual and organizational characteristics on employability competences and on internal and external employability. Research using multi-wave designs can help us to obtain more specific information about the stability and change of the variables, and about cross-lagged (i.e., over time) relationships (Spurk and Abele, 2014). A developmental approach to employability and consideration of the diversity of intra-individual change trajectories over time (Martin and Hofer, 2004; De Jonge and Dormann, 2006) might also be a very appealing approach for future research. Furthermore, both experimental and qualitative studies have the potential to advance our insight in the phenomenon of employability, and its inherent dynamism.

Second, as already stated in the “3 Methodology” section, there might be a common-source bias, which may have inflated correlations among the variables in our research. To properly address the possibility of CSB we conducted a Harman-1 factor analysis which showed no indication of inflated correlations in our data. Moreover, we used these data because employee perceptions and experiences are our key interest (George and Pandey, 2017).

### 5.2 Practical implications

Based on the results of this study, it is recommended for the management of public sector organizations to pay careful attention to monitoring and, where possible, further promoting the employability of all workers throughout their career. In order to prevent for a loss of employability competences as people grow older, their direct supervisors should effortfully invest in opportunities for maintaining the employability competences of their employees by adopting tailored strategies that preferably take into account a broader conceptualization of age [see Sterns and Doverspike (1989) who differentiated between chronological, organizational, functional, psychosocial, and life-span development age for more details]. After all, the development and further enhancement of employability competencies across the life-span requires that both the individual worker and their employer take into account that aging at work is a multi-dimensional process indicating changes in psychological, physical, social as well as societal functioning across time (De Lange et al., 2006; Kooij et al., 2008). Obviously, this process of aging at work has a profound impact on workers’ employability (De Lange et al., 2021).

In addition, the differences between men and women, differences between employees with different educational backgrounds and differences in the number of contract hours and contract type should also be taken into account. In doing so, individual customization can be carried out whereby HRM activities should be aimed at increasing those specific employability competences that require attention. We advocate a non-normative approach to this and to link up with the preferences, abilities possibilities and limitations of the employee, being the owner of his/her career as a person (Van der Heijden, 2005).

If one wants to influence the internal employability of employees, it is especially important to pay attention to the role of the supervisor. The more constructive (transformational) leadership employees experience, the more likely they are to consider themselves to be more internally employable. At the same time, it is important to reduce the amount of red tape in order to increase positive perceptions regarding one’s internal employability. Personality and risk-taking are more difficult to influence, although, as this study shows, they do have an influence on employability competences and on internal and external employability.

If external employability is to be influenced, it is particularly important to invest in occupational expertise and in the anticipation and optimization of employees. These competencies are partly dependent on the personality and risk-taking behavior of the employee and partly on the experienced (degree of transformational) leadership style and red tape.

## Data availability statement

Publicly available datasets were analyzed in this study. This data can be found here: https://easy.dans.knaw.nl/ui/datasets/id/easy-dataset:161487/tab/2.

## Ethics statement

The studies involving human participants were reviewed and approved by Erasmus Research Services, Secretary Research Ethics Review. The patients/participants provided their written informed consent to participate in this study.

## Author contributions

Both authors listed have made a substantial, direct, and intellectual contribution to the work, and approved it for publication.

## Footnotes

1
https://kennisopenbaarbestuur.nl/
